# Development and application of a *Bacillus anthracis* protective antigen domain-1 in-house ELISA for the detection of anti-protective antigen antibodies in cattle in Zambia

**DOI:** 10.1371/journal.pone.0205986

**Published:** 2018-10-18

**Authors:** Manyando Simbotwe, Daisuke Fujikura, Miyuki Ohnuma, Ryosuke Omori, Yoshikazu Furuta, Geoffrey Munkombwe Muuka, Bernard Mudenda Hang’ombe, Hideaki Higashi

**Affiliations:** 1 Division of Infection and Immunity, Research Center for Zoonosis Control, Hokkaido University, Sapporo, Japan; 2 Graduate School of Veterinary Medicine, Hokkaido University, Sapporo, Japan; 3 Asahikawa Medical University Education Research Promotion Center, Asahikawa, Japan; 4 Division of Bioinformatics, Research Center for Zoonosis Control, Hokkaido University, Sapporo, Japan; 5 Bacteriology Section, Central Veterinary Research Institute, Ministry of Fisheries and Livestock, Lusaka, Zambia; 6 Microbiology Unit, School of Veterinary Medicine, University of Zambia, Lusaka, Zambia; 7 Hokudai Center for Zoonosis Control in Zambia, Lusaka, Zambia; Spectrum Health, UNITED STATES

## Abstract

In Zambia, anthrax outbreaks among cattle are reported on nearly an annual basis. Presently, there is a lack of serological assays and information to develop an anthrax management and control strategy. In this study, an indirect enzyme-linked immunosorbent assay (ELISA) based on recombinant protective antigen domain 1 (rPA-D1) of *Bacillus anthracis* was developed and used to detect anti-PA antibodies in cattle in Zambia. An antigen coating of 10 ng/well and a serum dilution of 1:100 were determined to be the optimal rPA-D1 ELISA titration conditions. The intra- and inter-assay % coefficients of variation were less than 10% and 15%, respectively. The rPA-D1 ELISA could detect seroconversion in the cattle 1 month after anthrax vaccination. In a cross-sectional study conducted in the Western Province, Zambia, 187 serum samples from 8 herds of cattle were screened for anti-PA antibodies using the rPA-D1 ELISA. The seropositive rate of the serum samples was 8%, and the mean anti-PA antibody was 0.358 ELISA units. Additionally, we screened 131 cattle serum samples from Lusaka, which is a nonendemic area, and found no significant association between the antibody levels and sampling area (endemic versus nonendemic area). Conversely, significant differences were observed between the anti-PA antibody levels and herds, anti-PA antibody levels and vaccination status and anti-PA antibody levels and vaccination timing. Collectively, these findings suggest that the rPA-D1 ELISA is a useful tool for the detection of anti-PA antibodies in cattle in Zambia. The low proportion of seropositive sera indicates that there is inadequate cattle vaccination in the Western Province and, in addition to other epidemiological factors, this may precipitate the anthrax outbreak recurrence.

## Introduction

Anthrax is a fatal zoonotic disease caused by *Bacillus anthracis*. Anthrax continues to pose a serious public health and socioeconomic threat in several developing Asian and African countries. Occasionally, sporadic cases occur in the nonendemic areas and developed countries [1–3].

Cattle in Zambia are reared under the commercial and traditional farming sectors. Although the commercial sector rears exotic breeds (Freshian and Jersey), the traditional sector is characterized by subsistence farming, the management of indigenous breeds (Barotse and Angoni), and communal grazing. There are an estimated 3.0 million cattle in Zambia [4], with more than 80% present in the traditional farming sector. In Zambia, frequent anthrax outbreaks occur in cattle in the Western Province and occasionally in wildlife in the game management areas [5–7]. These outbreaks cause significant economic losses because a majority of the population is dependent on livestock for their livelihood and wildlife is a source of revenue for the government. In 2016, five anthrax outbreaks occurred in cattle in the Western Province, Zambia and one anthrax outbreak occurred in wildlife in the Chama District, Zambia [8, 9]. Over 60 cases in cattle and 18 cases in wildlife were reported. Furthermore, more than 100 suspected human cases were caused by exposure from the handling and consumption of the contaminated meat of infected cattle and wildlife [8, 10].

Previous studies have demonstrated that the ecology of *B*. *anthracis* is influenced by the climatic and environmental factors [11]. In Zambia, the recurrence of a livestock anthrax outbreak has been closely linked to seasonal variations and human dependency on the Barotse Floodplain [12, 13]. Despite the available information on the epidemiology of anthrax in Zambia, it remains difficult to evaluate the susceptibility of cattle and vaccination coverage due to under-reporting and the remoteness of the affected areas. Furthermore, much of what is known about serology of anthrax in the endemic areas elsewhere is largely based on the studies in wildlife [14–16], and the serology of anthrax in cattle has thus been a neglected area of anthrax surveillance. In this regard, the detection of antibodies against *B*. *anthracis* in cattle in endemic areas is important to understand the present seroepidemiologic situation and its contribution to anthrax outbreak recurrence.

At present, Zambia has a passive surveillance system that is mostly functional following the reports of suspected anthrax cases. The reporting system in the Western Province comprises three administrative levels: veterinary camp, district levels and provincial levels. After a suspected case of anthrax has been identified, samples are collected and sent to the provincial veterinary office where Giemsa staining is performed; thereafter, the positive samples are sent to Lusaka (Lusaka Province) for confirmatory tests. In Lusaka, bacterial culture and isolation are performed. It is sometimes difficult to confirm the diagnosis through the sample cultures from suspected anthrax cases that are undergoing antibiotic treatment. Since antibiotics do not eliminate the anthrax toxin components or other components of the bacteria, using a serological assay to detect antibodies to toxin components would complement the present methods being used in anthrax surveillance.

The virulence of *B*. *anthracis* is caused by the anthrax toxin and antiphagocytic capsule. Genes encoding the anthrax toxin are located on the pXO1 plasmid [17, 18] and the cap region which is important for encapsulation is located on the pXO2 plasmid [19–21]. Anthrax toxin comprises three proteins namely: protective antigen (PA), edema factor and lethal factor. Because PA is the superior antigen in the natural and vaccine-induced immunity to anthrax infection [22, 23], the PA-based enzyme-linked immunosorbent assay (ELISA) is presently the most effective method for detecting antibodies against *B*. *anthracis* in serum, plasma or other biological fluids. It is sensitive and enables hundreds to thousands of samples to be screened in a short period of time. Certain advances have been made in the ELISA mainly based on full-length PA, and the ELISA has been applied to human anthrax vaccine evaluation and diagnosis [24, 25]; however, it still has certain drawbacks such as false positives, lack of robustness and lack of assay standardization. Few studies have assessed the development and use of ELISAs in the detection of anti-PA antibodies in livestock populations [16, 26, 27]. In these studies, the commercial ELISA kits were primarily used in developed or nonendemic countries for the evaluation of an antibody response after the anthrax vaccination in livestock under commercial settings. Additionally, the commercial assays are unsuitable for use in resource-limited settings, where it is difficult to maintain cold chain facilities for the storage of antigens or ELISA precoated plates. Thus, a sensitive, specific and robust ELISA for detecting antibodies against *B*. *anthracis* PA in cattle in resource-limited settings is necessary.

PA has four domains: domains 1, 2, 3 and 4 [28]. Each domain has a specific function in the host cell intoxication [29]. PA domain 1 (PA-D1) contains the furin cleavage site RKKR and proteolytic cleavage is required for the activation of anthrax toxin [30]. PA-D1 also contains the Ca^2+^ binding sites that provide structural stability to PA [31]. PA-D1 is immunogenic and its qualitative immune response has been shown to be equivalent to that elicited by whole PA [32, 33]. Immunoassays based on PA or its domain for the detection of anti-PA antibodies in the serum of livestock in endemic areas may provide useful information on the anthrax exposure patterns and the effectiveness of anthrax vaccination programs. Previous studies have suggested the potential use of PA domains in the development of serodiagnostic assays for anthrax [34]; however, an ELISA based on a subunit of PA has not yet been developed.

To overcome the above limitations and to demonstrate the utility of a subunit of PA, we developed a qualitative bovine ELISA using recombinant PA-D1 (rPA-D1) as an ELISA antigen. Furthermore, to demonstrate the practical use of the rPA-D1 ELISA, we screened sera collected from cattle herds from the Western Province, Zambia for antibodies against *B*. *anthracis* PA.

## Materials and methods

### Expression and purification of rPA-D1

PA-D1 is composed of residues 1–258. The following primers were designed and then synthesized by Sigma Aldrich: PA-D1 forward primer (5′-ATG GAA GTT AAA CAG GAG AAC CGG-3′) and PA-D1 reverse primer (5′-TGC CAC AAG GGG GTG TCT TG-3′). The gene encoding PA-D1 was amplified from *B*. *anthracis* CZC5 (accession number: AP018443) by TaKaRa Ex Taq. The amplified PCR products were analyzed on 1% agarose gel and extracted using the QIAquick Gel Extraction Kit (Qiagen). The purified fragments were then subjected to cloning experiments with the pGEM-T vector system (Promega) and *Escherichia coli* DH5 α. Plasmid DNA was purified by a QIAprep Spin Miniprep Kit (Qiagen). The pGEMT-D1 and pGEX6p2 plasmids were then digested with the restriction enzymes BamHI and NotI (New England BioLabs). The PA-D1 gene was ligated into the BamHI and NotI sites of the expression vector pGEX6p2 (pGEX6p2-PA-D1).

*E*. *coli* BL21 cells were transformed with the expression vector pGEX6p2-PA-D1. The transformed *E*. *coli* BL21 cells were grown at 37°C for 15 h at 180 rpm in 3 ml of Lysogeny broth supplemented with 100 μg/ml of ampicillin. The culture was then added to 70 ml of Terrific Broth (TB) and the cells were grown at 37°C for an additional 2 h at 180 rpm. After reaching an OD_600_ of 1.8, the culture was diluted until it reached an of OD_600_ 0.05 in 1 L of TB. The cells in the 1 L of culture were grown at 37°C for 90 min at 80 rpm and then at 15°C for 30 min. When the OD_600_ reached 0.8, protein expression was induced by adding isopropyl *β*-D-thiogalactopyranoside (IPTG) to reach a final concentration of 1 mM; thereafter, the cells were grown at 18°C for 20 h. The cell culture was centrifuged at 4,000 rpm for 15 min and the supernatant was extracted. The resulting cell pellet was suspended in lysis buffer [phosphate buffered saline (PBS) containing 0.5% Triton X-100, 0.1 mM benzamidine, 1 mM PMSF, pH 7.5]. The resuspended cell pellet was centrifuged for 15 min at 4,000 rpm, supernatant removed and stored at −80°C. The supernatant fraction (containing the soluble fusion protein) was extracted from the frozen *E*. *coli* cell pellet. Briefly, the cell pellet was resuspended in lysis buffer and whole cells were lysed using a pressure cell homogenizer (Stansted Fluid Power). The resulting suspension was centrifuged at 4°C for 30 min at 15,000 rpm. The supernatant was treated with 50% Glutathione Sepharose 4 beads (GE Healthcare) using the manufacturer's protocol for batch purification of GST-tagged proteins. The GST tag of the protein bound to the resin was cleaved by a PreScission Protease (GE Healthcare) reaction for 16 h at 4°C. The recombinant protein was eluted using elution buffer (50 mM Tris, 150 mM NaCl, 1 mM EDTA, 1 mM DTT, pH 7.5). The eluted fraction was then filtered using a 0.22-μm filter unit (Corning) and loaded onto a HiPrep 26/10 desalting column (GE Healthcare) for buffer exchange. After the desalting process, the collected fraction was refiltered and loaded onto a Resource-S column (GE Healthcare) equilibrated with 25 mM MES, pH 6.5. A NaCl gradient was used for the elution. The purified rPA-D1 protein was stored in aliquots at −80°C. Fractions at all stages of the expression and purification process were analyzed by sodium dodecyl sulfate polyacrylamide gel electrophoresis (SDS-PAGE, 12%) and Western blot. For the Western blot analyses of the rPA-D1 protein, GST Mouse Monoclonal IgG (Santa Cruz) was used as a primary antibody and anti-mouse IgG-HRP (GE Healthcare UK Limited) was used as a secondary antibody. rPA-D1 was further treated with PAb anti-*B*. *anthracis* protective antigen (Norvus) and anti-Rabbit IgG (GE Healthcare UK Limited). All steps of the purification process were performed at 4°C. The purified rPA-D1 protein was checked for residual lipopolysaccharide (LPS) content using the Limulus amebocyte lysate (LAL) chromogenic assay (Endospecy ES-50M Kit; Seikagaku).

### rPA-D1 ELISA

An indirect ELISA format was used. Titration tests were performed to determine the optimal concentrations of the reagents. Briefly, 96-well flat-bottomed microtiter plates (Corning) were coated with 100 μl per well of diluted rPA-D1 at a concentration of 100 ng/ml in (PBS, pH 7.4) overnight at 4°C. The coated plates were manually washed thrice (300 μl per well) with a wash buffer (PBS containing 0.05% Tween-20) and were blotted dry on paper towels. The wells were then blocked with 100 μl of blocking buffer (5% skim milk in PBS containing 0.05% Tween-20, pH 7.4). After washing, the serum samples were diluted to 1:100 in blocking buffer and then 100 μl of the diluted solution was added to wells in triplicate and incubated for 1 h at room temperature (RT). After another washing step, the Rabbit anti-Bovine IgG-HRP (Invitrogen) was diluted to 1:12,000 in blocking buffer, and 100 μl of the diluted antibody solution was added to each well and incubated for 1 h at RT. After the final washing step, the bound secondary antibody was detected by adding 100 μl of TMB substrate (KPL) per well. Color development was observed after 20 min of incubation at RT. Color development was stopped by the addition of 50 μl of 1 N sulfuric acid per well. Absorbance values were read using a microtiter plate reader (Thermoscientific) at a wavelength of 450 nm (OD_450_). OD_450_ values of the test sera were expressed in ELISA units (EUa). Each plate contained the negative control (NC) and positive control (PC) sera. The mean OD_450_ of the three blank wells was subtracted from each OD_450_ value of the test serum sample and the result expressed as a proportion of the PC.

The rPA-D1 ELISA cut-off value at OD_450_ was determined as follows: the mean of the NC group (*n* = 53) + 3 standard deviations (mean + 3 SD). Samples with OD_450_ values greater than the cut-off were considered positive. Coefficients of variation (CVs) within an assay plate and between different assay plates were used to assess the reproducibility of the rPA-D1 ELISA. To evaluate the intra-assay variation, replicates of the control sera were run within a plate; to evaluate the interassay variation samples were run on different occasions. The CV was calculated using the following formula: CV = SD/mean × 100%.

### Study area

**Fig 1:**
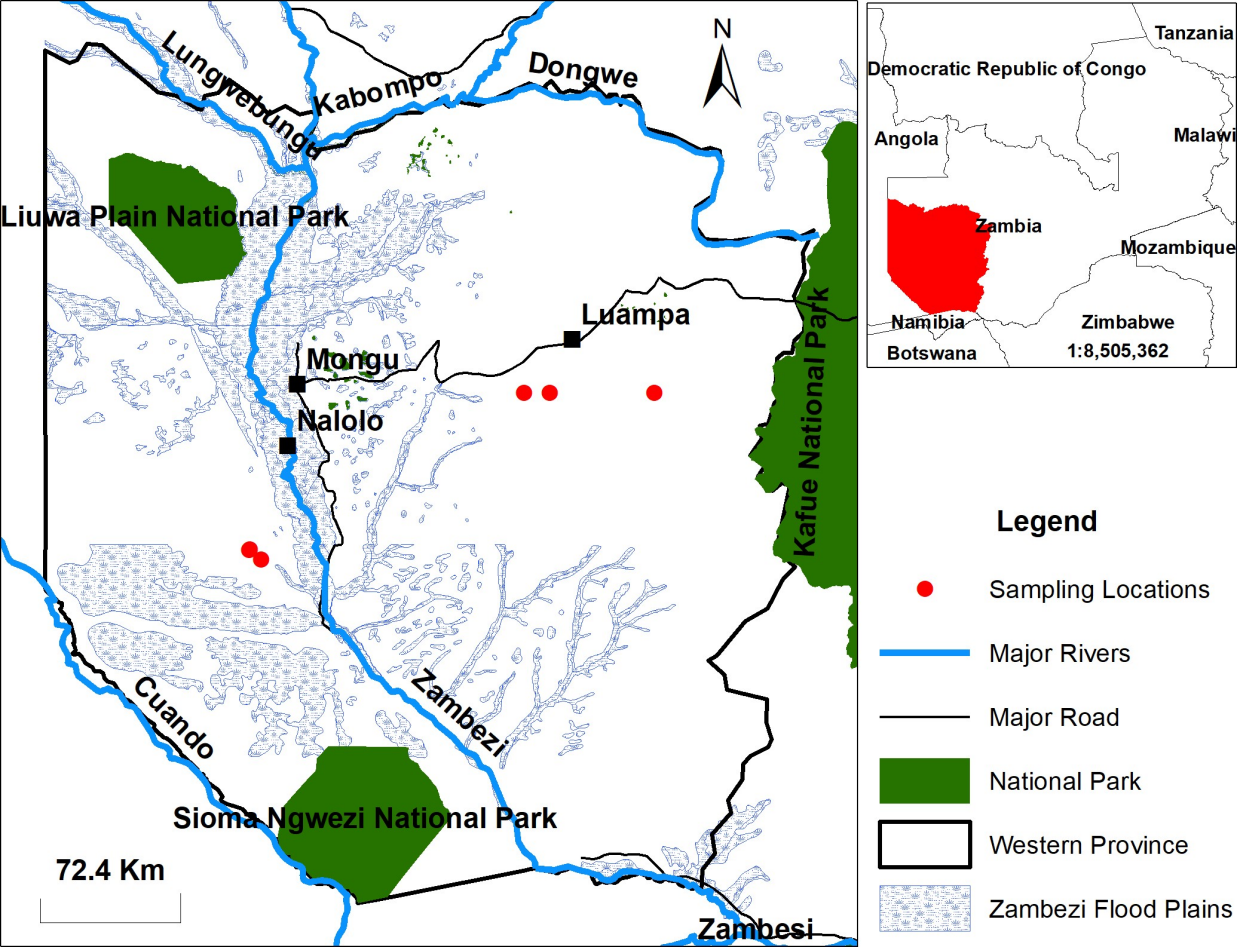
Sampling areas in the Western Province, Zambia. The blood samples used in this study were collected from eight herds of cattle in the Nalolo District and Luampa Districts.

Blood samples were collected from the Kaunga Lueti and Luampa veterinary camps which cover two herds in the Nalolo District (*n* = 42, 16° 01.417'S, 22° 55.976'E and 16° 01.252'S, 22° 56.124'E), and six herds in the Luampa District (*n* = 145, 15° 18.354'S, 24° 11.331'E, 15° 15.961'S, 24° 18.281'E and 15° 37.229'S, 24° 46.901'E) of the Western Province, Zambia (Fig 1). Farmers in these areas practice the transhumance type of livestock farming which is characterized by the seasonal movement of cattle and communities out of the floodplain to the upland areas. The plains are important because they provide grazing pasture for the cattle and influence the livelihood and culture of its inhabitants. The Western Province sampling sites were chosen based on the history of anthrax outbreaks obtained from the district veterinarians. In this study, the Western Province is referred to as an endemic area because anthrax outbreaks are reported almost annually here. Samples were collected in Lusaka Province (*n* = 131, 15°25'S, 29°0'E). The nonendemic area, Lusaka, refers to an area with no anthrax outbreak reports for more than 20 years. An anthrax case was suspected when there was a sudden death and the epidemiological history indicated reports of previous outbreaks in that location. In the cases in which the carcasses were opened, extravasation of blood from orifices and an enlarged spleen were observed.

### Cattle test sera

Approximately 5 ml of blood was collected in sterile plain blood collection tubes and stored at 4°C until serum sample preparation. The serum samples were prepared by allowing the collected blood samples to stand at RT for 1 h. The samples were then centrifuged at 25°C for 15 min at 3,000 rpm. The serum samples were aliquoted into clean microcentrifuge tubes and were stored at −20°C. The samples were then analyzed at the Hokudai Center for Zoonosis Control, School of Veterinary Medicine in Zambia.

A total of 398 bovine serum samples were used in this study. NC group (*n* = 55): The NC group included a total of 29 serum samples that were obtained from Lusaka from cattle with no known history of anthrax vaccination and 26 serum samples that were obtained from unvaccinated calves in herds with a history of anthrax vaccination in Lusaka. Of the 55 samples, 53 were used to determine the ELISA cut-off value. PC group (*n* = 25): The PC group included serum samples (*n* = 24) that were obtained from cattle one month after the anthrax vaccination and one pooled serum sample that was obtained from cattle with naturally acquired anthrax infection. Endemic area (*n* = 187): The endemic area group included serum samples that were obtained from eight herds of cattle in the Western Province. Nonendemic area (*n* = 131): The nonendemic area included serum samples that were obtained from cattle in the Lusaka Province.

### Statistical analyses

We calculated the symptom-based prevalence by dividing the number of suspected anthrax cases in a herd by the number of cattle in that herd. The mortality rate was calculated by dividing the number of deaths in a herd by the number of cattle in that herd. For the statistical analysis, we initially tested whether the data followed a normal distribution using the Shapiro–Wilk test. Comparisons of the means of anti-PA antibodies of the herds were performed using the Kruskal–Wallis test. To test the significance of the association between anti-PA antibodies and various states (vaccination timing and epidemic status), we performed the Wilcoxon rank sum test. Graphs were generated using computer graphics software (GraphPad Prism, version 7.0b; GraphPad, San Diego, CA) and R software version 3.3.3 [35]. A *p* value <0.05 was considered as statistically significant.

### Ethical statement

Ethical clearance was obtained from the ERES Converge Review Board, Lusaka (Ref. No. 2017-Apr-006). Permission to conduct sampling was obtained from the Ministry of Fisheries and Livestock (MFL), Zambia. Before collecting the blood samples from various cattle herds, meetings were held with the farmers and community leaders to explain the purpose of the study, to obtain a brief history of anthrax vaccination and to obtain informed verbal consent. Blood samples were collected in accordance with the regulations for animal husbandry in Zambia.

The ERES Converge Review Board, Lusaka, approved both the data collection from cattle as and the data collection though interviews/discussions with farmers and community leaders. No formal documentation was required when obtaining oral consent. Obtaining written consent was not a requirement as per the Ethics Committee that approved this study.

## Results

### Antigen preparation

**Fig 2:**
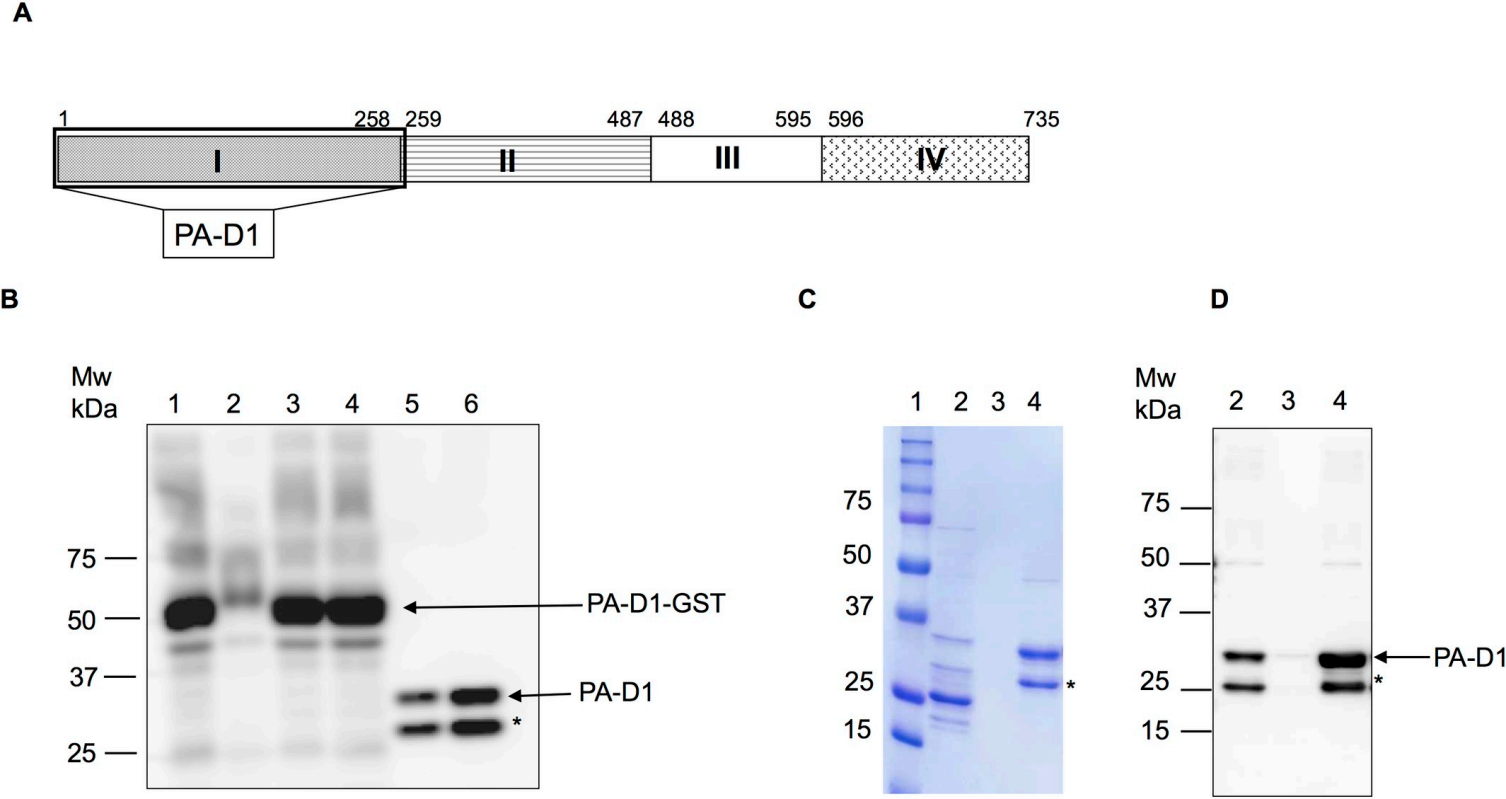
Production and purification of rPA-D1. (A) Cartoon of full-length PA revealing the PA-D1 gene used in antigen preparation. (B) Western blot analysis of the affinity purification and desalting process: lane 1, total cell lysate; lane 2, pellet fraction; lane 3, supernatant fraction (PA-D1-GST: 54 kDa); lane 4, beads bound; lane 5, elute after treatment with PreScission Protease (PA-D1: 28 kDa); lane 6, desalting fraction. (C and D) Coomassie brilliant blue staining and Western blot analysis of the fractions collected during the cation exchange process. Lane 1, molecular weight marker; lane 2, sample loaded onto the cation exchange chromatography column; lane 3, flow through; lane 4, Resource-S fraction. Mw; molecular weight marker (in kDa), *: truncated product.

PA-D1, one of the four domains of PA, is crucial in mediating the anthrax toxin entry into the host cell. To prepare rPA-D1 (Fig 2A) as an ELISA antigen, rPA-D1 was expressed as a GST fusion protein in *E*. *coli* and was clarified in the supernatant fraction of the cell lysate. Before and after the GST tag cleavage, a GST-rPA-D1 band of approximately 54 kDa and rPA-D1 of 28 kDa were detected (Fig 2B). Following the affinity purification process, rPA-D1 was subjected to further purification via ion exchange chromatography. Coomassie brilliant blue staining and Western blot analysis were used to confirm the purity of the collected protein fractions (Fig 2C and 2D). From 1 L of culture, approximately 0.8 mg of rPA-D1 was obtained with a purity of more than 90%. The LPS content of the rPA-D1 protein used in the ELISA was determined as 0.02 endotoxin units (EUb) /ml using the LAL assay.

### Development of rPA-D1 ELISA

**Fig 3:**
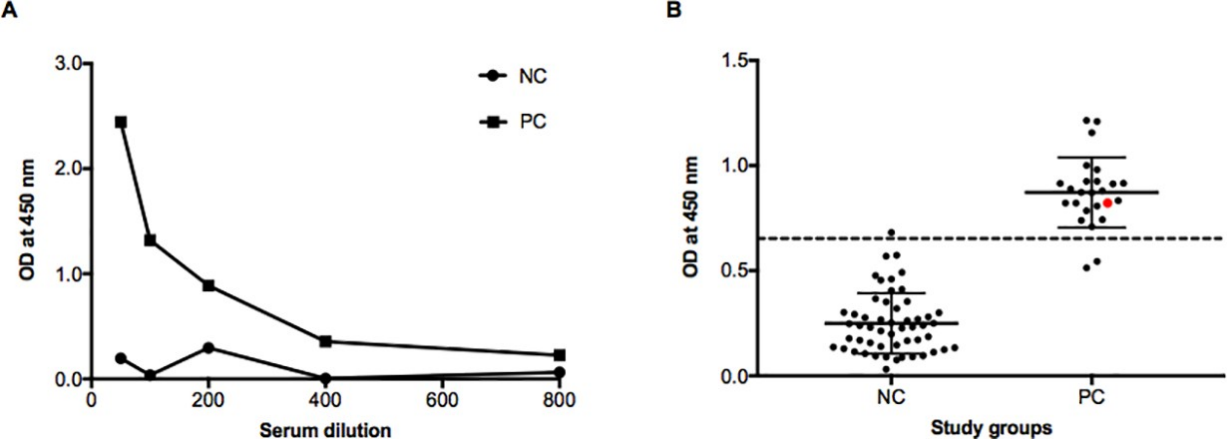
Optimization of rPA-D1 ELISA. (A) By using the checkerboard titrations, we determined the optimal concentrations of the antigen, the antibody and serum dilutions as follows: antigen, 10 ng/well; serum dilution, 1:100; second antibody dilution, 1:12,000. (B) Analysis of control serum samples using rPA-D1 ELISA under optimal conditions. NC: negative control group, (*n* = 53). PC: positive control group (*n* = 25). Red dot: pooled sample from cattle with exposure to natural infection. Error bars represent the mean and standard deviation. Horizontal dotted line: cut-off value of 0.67.

To determine the optimal ELISA conditions and concentrations of reagents, we performed checkerboard titrations. An antigen concentration of 100 ng/ml, a serum dilution of 1:100 and a secondary antibody dilution of 1:12,000 were determined as the optimal titration conditions (Fig 3A). To determine rPA-D1 stability and use in the absence of cold chain facilities, we compared the use of rPA-D1-precoated ELISA plates with lyophilized rPA-D1 stored at RT. After 3 weeks of storage, we found that the use of freshly prepared plates using lyophilized rPA-D1 was superior to that of the rPA-D1-precoated plates (S1 Fig). Moreover, we compared the sera from herds in the NC group (unvaccinated cattle with no history of vaccination versus unvaccinated calves from a herd with a history of vaccination) used to determine the cut-off value and found no significant difference between the groups (Mann–Whitney U test, *p* > 0.05). The rPA-D1 ELISA cut-off value was determined as the mean of the NC group + 3 SD. The OD_450_ values of the NC group (*n* = 53) ranged from 0.03 to 0.68. The mean of the NC group was 0.249 and the SD was 0.143; and the cut-off value was thus determined to be 0.678 (Fig 3B). To validate the repeatability of the rPA-D1 bovine ELISA, we calculated the intra- and interassay CVs of the NC and PC serum samples. The intra-assay CV of PC was 5.5% (*n* = 10 replicates, 0.3%–13.2%) and that of NC was 4.5% (*n* = 9 replicates, 0.3%–12.7%). The interassay CV of PC was 4.13% (*n* = 11 replicates, 0.58%–10.4%) and that of NC was 8.1% (*n* = 10 replicates, 4%–16.4%). The CVs were within the reference ranges of ≤ 10% and ≤15%, respectively.

### Herd history

Information on the history of the herds was obtained through meetings with district veterinarians, livestock farmers and community leaders. We found that between November 2013 and January 2014, there was an anthrax outbreak that affected herds 1–8 in the Nalolo District and Luampa Districts (Table 1). In response to these outbreaks, the district veterinarians performed vaccinations, burying and decontamination with lime. In some villages, the carcasses were consumed. At the time of sample collection in October 2016, the cattle had not been revaccinated since the 2013–2014 anthrax outbreak. We calculated the symptom-based prevalence and mortality rates in eight herds of cattle based on the number of suspected cases and mortalities recorded during the outbreak. The symptom-based prevalence among herds ranged from 0% to 36.4% with an average of 15.3%. The average herd symptom-based prevalences in the Nalolo District and Luampa Districts were 21.7% and 13.9%, respectively, and the mortality rates in the two districts were 15.2% and 5.1%, respectively. The average herd mortality rate was 6.9% (Table 1).

**Table 1:** Herd history.

					2013–2014 anthrax outbreak				
Herd ID	Camp	Crush pen	Village	Herd size	Date of outbreak^a^	Vaccination status prior to outbreak	Symptom-based prevalence (%)	Mortality (%)	Control measures taken
1	Luampa	Mbundu	Muzangabantu	13	Nov-2013	Not vaccinated	0 (0.0)	0 (0.0)	Vaccination
2	Luampa	Mbundu	Muzangabantu	80	Nov-2013	Not vaccinated	20 (25.0)	4 (5.0)	Vaccination
3	Luampa	Mbundu	Muzangabantu	37	Nov-2013	Not vaccinated	0 (0.0)	0 (0.0)	Vaccination
4	Luampa	Pulobende	Mwembwe	32	Nov-2013	Not vaccinated	4 (12.5)	2 (6.3)	Burying and vaccination
5	Luampa	Nakayembe	Sikabilima	37	Nov-2013	Not vaccinated	4 (10.8)	2 (5.4)	Burying and vaccination
6	Luampa	Pulobende	Mwembwe	17	Nov-2013	Not vaccinated	2 (11.7)	3 (17.6)	Vaccination
7	Kaunga lueti	Malombe	Kaonga	35	Jan-2014	Not vaccinated	6 (17.1)	5 (14.2)	Burying
8	Kaunga lueti	Malombe	Kaonga	11	Jan-2014	Not vaccinated	4 (36.4)	2 (18.1)	Burying and vaccination

^**a**^Dates; month-year.

Fascioliasis and Black quarter are the common illnesses that usually affect cattle. Livestock owners generally administer broad spectrum antibiotics (oxytetracycline and penicillin) to treat any sick animals in the herds.

### Serology

A total of 318 serum samples were screened for anti-PA antibodies using rPA-D1 ELISA. Of the 187 samples from the endemic area, only 8% (95% confidence interval (CI): 4.1–11.9%) were positive (Table 2). Herd 8 revealed majority of positive samples (22.2%), whereas no seropositive samples were detected in Herd 3 and 5. The seropositivity of the samples from the nonendemic area was 18.3% (95% CI: 11.7–24.9). Seropositivity ranged from 0% to 22.2% in the endemic area and it ranged from 5.7% to 42.8% in the nonendemic area. The mean OD_450_ of the serum samples from the endemic and nonendemic area were 0.358 (95% CI: 0.328–0.388) and 0.452 (95% CI: 0.406–0.499), respectively (Table 2).

**Table 2:** Proportion of seropositive cattle in the herds sampled.

Sampling area	Herd ID	When last known anthrax case was detected	When last vaccinated before sampling^a^		Date of sample collection^a^	Mean (SD) anti-PA Ab (EUa)	No. of samples tested	No. positive	% positive (95% CI)
**Western Province**	1	No cases	Dec-2013		Oct-2016	0.428 (0.292)	11	2	18.2 (−4.6, 41.0)
	2	Nov-2013	Dec-2013		Oct-2016	0.277 (0.181)	39	1	2.6 (−2.4, 7.5)
	3	No cases	Dec-2013		Oct-2016	0.270 (0.138)	32	0	0 (0.0, 0.0)
	4	Nov-2013	Jan-2014		Oct-2016	0.454 (0.201)	22	3	13.6 (−0.7, 28.0)
	5	Nov-2013	Jan-2014		Oct-2016	0.319 (0.150)	25	0	0 (0.0, 0.0)
	6	Nov-2013	Jan-2014		Oct-2016	0.396 (0.212)	16	2	12.5 (−3.7, 28.7)
	7	Jan-2014	May-2015		Oct-2016	0.427 (0.217)	33	5	15.2 (2.9, 27.4)
	8	Jan-2014	May-2015		Oct-2016	0.495 (0.259)	9	2	22.2 (−4.9, 49.4)
**Total**						0.358 (0.207)	187	15	8 (4.1, 11.9)
**Lusaka Province**	9	No cases	NA		Oct-2016	0.744 (0.268)	21	9	42.8 (21.7, 64.0)
	10	No cases	Nov-2015		Apr-2017	0.430 (0.196)	28	4	14.2 (1.3, 27.3)
	11	No cases	Oct-2014		Oct-2016	0.254 (0.173)	35	2	5.7 (−2.0, 13.4)
	12	No cases	Nov-2015		Sep-2016	0.636 (0.209)	23	7	30.4 (11.6, 49.2)
	13	No cases	Oct-2014		Oct-2016	0.335 (0.167)	24	2	8.3 (−2.7, 19.4)
**Total**						0.452 (0.268)	131	24	18.3 (11.7, 24.9)

^**a**^Dates; month-year.

NA; not available.

**Fig 4:**
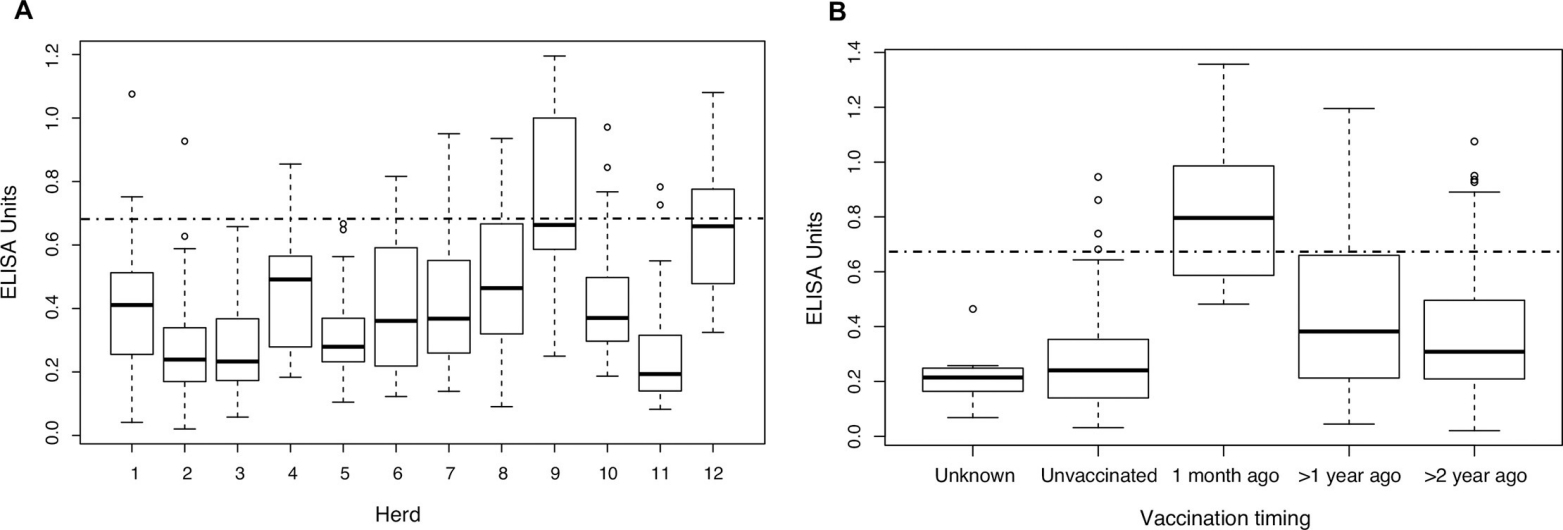
Association between the anti-PA antibodies and state. Box plots for the distribution of anti-PA antibodies. (A) Herds 1–8 in the endemic area and 9–12 in the nonendemic area. (B) Vaccination timing. The boxes indicate the interquartile range, the horizontal lines indicate the median, and the lower and upper hinges indicate the minimum and maximum values, respectively (outliers not included). Horizontal dotted line: cut-off value of 0.67.

We used the Kruskal-Wallis test to compare the means of anti-PA antibodies in different herds and found a significant difference in the means of anti-PA antibodies of herds (*p* < 0.05). Since most of the herds in this study were previously exposed to vaccination, we compared the distribution and residual levels of anti-PA antibodies according to the epidemic status. We found no significant difference between the anti-PA antibodies of cattle in the endemic area and the nonendemic area (*p* > 0.05). The overall residual level of anti-PA antibodies in herds in the endemic area was comparable to that in herds in the nonendemic area (Fig 4A). We then analyzed the association between the antibodies and vaccination timing using the Wilcoxon rank sum test. We found significant differences between the unvaccinated and vaccinated herds. A significant difference was observed between the anti-PA antibodies and vaccination timing, specifically 1 month ago displayed significantly higher anti-PA antibodies compared to >1 year ago, 1 year ago displayed significantly higher anti-PA antibodies compared to 2 years ago (*p* < 0.05). One month and >1 year ago displayed significantly higher anti-PA antibodies compared to the unvaccinated group (*p* < 0.05). Conversely, >2 years ago was not significantly different from the unvaccinated group (*p* > 0.05; Fig 4B).

## Discussion

Serological assays for anthrax epidemiological investigations are currently unavailable in Zambia. Serological testing is necessary not only for diagnostic purposes but also for determining the effectiveness of vaccination programs and obtaining epidemiological information in anthrax endemic areas. In this study, we developed an indirect qualitative bovine ELISA using rPA-D1 of *B*. *anthracis* as an antigen and demonstrated its utility by screening the cattle herds for antibodies against *B*. *anthracis* PA in the Western Province, Zambia.

In accordance with the previous observations, rPA-D1 was prone to degradation during the purification process; however, that had no effect on the ELISA setup and optimization [33]. The residual content of LPS in the rPA-D1 protein used for ELISA plate coating was less than 0.05 EUb/ml according to the LAL assay. Although we observed low background noise due to the rPA-D1 protein and other bacterial contaminants during our assay optimization and sample analyses, there is a possibility that this LPS content may have influenced some of our results.

Our rPA-D1 ELISA revealed high levels of reproducibility with CVs of less than 10% and 15%, respectively. The ELISA was able to detect seroconversion in cattle 1-month postvaccination. Some serum samples in the NC group had high OD_450_ values. This may be attributed to passive immunity, since some serum samples were collected from the unvaccinated calves in a herd that was previously exposed to anthrax vaccination. To determine whether the sera from this group were increasing the mean OD_450_ readings of the NC group, we compared the two groups (unvaccinated cattle with no history of vaccination versus unvaccinated calves from a herd with a history of vaccination) used to determine the cut-off value; however, we found no significant difference between the groups (*p* > 0.05). The group containing sera from the unvaccinated calves did not increase the mean OD_450_ of the NC group. Conversely, of the 25 positive samples, 2 had OD_450_ values below the cut-off value. This finding occurred because these cattle may have developed insufficient immunity after anthrax vaccination. Our results further substantiate the previous findings from which it was concluded that there is a variation in seroconversion in the individual animals and that time is essential to develop adequate immunity after vaccination; for example, following an anthrax outbreak in cattle in Australia, 132 cases of anthrax occurred 10 days after vaccination and 3 cases occurred 25–30 days after vaccination [36, 37]. Since a single cut-off value based on our NC group sera was assigned to the rPA-D1 ELISA, there may be some degree of false positive and negative results, in particular considering the history of the control group. In future works, the use of control sera obtained from herds with no history of exposure to vaccination and anthrax would enable us to estimate the sensitivity and specificity of this assay.

Although the performance characteristics of most ELISAs used in the serological studies of anthrax in livestock and wildlife have not been reported, we noted a few variations between these assays and the rPA-D1 ELISA, including differences in antigen preparation, antigen purity and the ELISA plate preparation process. For example, Turnbull [38] used 20 μg/ml of whole PA purified from cell-free filtrates of the Sterne strain of *B*. *anthracis* and used precoated ELISA plates. Conversely, we used 100 ng/ml of rPA-D1 and found that freshly prepared plates using lyophilized rPA-D1 stored at RT were superior to the precoated plates stored at RT. In addition, rPA-D1 ELISA eliminates the risk of having nonreactive wells, likely due to the denaturation of precoated capture antibody antigen complexes by using freshly prepared ELISA plates. Moreover, the use of a lyophilized antigen is ideal because it is difficult to maintain cold chain facilities in Zambia. The rPA-D1 antigen is safe and can be readily prepared. Therefore, the rPA-D1 ELISA could be used for detection of anti-PA antibodies in bovine serum after *B*. *anthracis* exposure or vaccination.

Our findings regarding herd history corroborate with the results of epidemiological studies conducted in the Western Province, Zambia [12, 39]. Previously, people have usually resorted to culling very sick cattle in the herd and consuming the meat. Additionally, the meat from infected carcasses or animals that died of unknown causes was consumed due to the assumed low risk of acquiring anthrax infection among the people in rural communities. Notably, Fascioliasis and Clostridial infections were still among the most common conditions that affected the animal health [40], and farmers reported that they administered broad spectrum antibiotics to treat sick animals. In our study, the average herd mortality rate was 7%, with a range from 0% to 18%. A similar herd mortality rate was reported during an anthrax outbreak in the Sesheke District [41], suggesting that the impact of anthrax does not necessarily depend on the proximity to parts of the Barotse Floodplain along Zambezi River, which are considered to be the stable natural anthrax foci.

Our findings regarding anthrax outbreak management and control during the November 2013 to January 2014 anthrax outbreak are consistent with measures taken in past outbreaks both in Zambia and elsewhere [42, 43]. Herds 1–6 experienced their first anthrax outbreak in November 2013. Prior to this outbreak, these cattle herds had no history of anthrax vaccination. Furthermore, during blood sampling in October 2016, we found that the cattle had not been revaccinated since the 2013 outbreak. Our findings suggest that there was a spread of infection from active anthrax foci. This is supported by the serological data (Table 2) and a previous study that reported that anthrax outbreaks were limited to the Barotse Floodplain [12].

To demonstrate the usefulness of the rPA-D1 ELISA, we screened the cattle sera collected from the anthrax endemic and nonendemic areas in Zambia. Although there was no ongoing outbreak in the Western Province at the time of sampling in October 2016, sampling was performed in October because the livestock anthrax outbreaks usually peak during the dry months of September–November. We expected that cattle in the endemic area may be exposed and that we would detect a high proportion of seropositive individuals. In contrast, we found that the overall proportion of seropositive sera from cattle in the Western Province was 8% (15/187). Since these herds were once exposed to anthrax vaccination, presumably we detected the residual antibodies from anthrax vaccinations. Collectively, with herd history, the results suggest that there is inadequate vaccination of cattle against anthrax in the study area.

Naturally acquired antibodies against anthrax have been reported in wildlife [44]. In serological studies of anthrax in wildlife, animals and humans, it was found that naturally acquired antibodies against anthrax were rarely found in herbivores, and that animals in the endemic areas have a high residual level of antibodies [16]. Conversely, in this study, we found an insignificant difference between the anti-PA antibody levels of cattle in the endemic and nonendemic area. Overall, the residual level of anti-PA antibodies in cattle in the endemic area was similar to that in cattle in the nonendemic area. Although most of the herds revealed low seropositive rates, we were able to detect a significant difference between the vaccinated and unvaccinated cattle and vaccination timings; we also observed a decrease of anti-PA antibodies over time (Fig 4B). These findings further validate the effectiveness of the rPA-D1 ELISA. Interestingly, we found no significant differences between cattle that were last vaccinated >2 years ago and the unvaccinated group. The detection of higher numbers of seropositive individuals in herds that were last vaccinated >2 years ago may indicate exposure to natural infection. We could not rule out the exposure of cattle to natural infection in this study; therefore, we equally found it difficult to make inferences concerning the exposure to natural infection because we collected samples at one-time point in the absence of an outbreak and the herds were once exposed to vaccination. During sampling, we did identify some herds with no history of vaccination; however, we were unable to collect samples from those herds because the livestock owners did not give consent. Further serological studies in the Western Province in herds without a history of anthrax vaccination or anthrax outbreaks would provide more insights on anthrax exposure in Barotse cattle.

Anthrax outbreaks in the endemic systems have been linked to the ecological factors, environmental, or reproductive stress factors [45]. Our results provide evidence that, in addition to the aforementioned factors, the outbreak occurrence is mainly due to inadequate vaccination. Although protection conferred by a single dose of livestock anthrax vaccine has been reported to be sufficient for one year [46], anthrax outbreaks have been reported in the vaccinated herds [36]. In this study, we detected a few seropositive sera from cattle in a herd in a nonendemic area 9 months after vaccination. The low seropositivity could be caused by factors such as batch quality in vaccine production and variations in the maintenance of cold chain facilities, although we cannot exclude the possibility that this finding is due to variability in the efficacy of anthrax vaccine (34F2 Sterne strain) in the field. Hence, further work is necessary to evaluate the present immunization schedule and duration of immunity conferred by the anthrax vaccines used in Zambia.

This study provides evidence that due to inadequate vaccination, the low proportion of seropositive cattle may be a factor contributing to anthrax outbreak recurrence in the Western Province, Zambia. The rPA-D1 ELISA will be a useful tool not only in surveillance but also in monitoring the effectiveness of anthrax vaccination programs in Zambia.

## Supporting information

S1 Fig Comparison of rPA-D1 precoated and freshly prepared ELISA plates. Comparison of the use of a freshly prepared ELISA plate using rPA-D1 stored at −80°C to that of a rPA-D1 precoated ELISA plate stored at RT and freshly prepared ELISA plate using lyophilized rPA-D1 stored at RT. Each PC and NC sample was measured in triplicate. The error bars represent the standard deviation. RT: Room temperature, PC: Positive control, NC: Negative control bovine serum.
(TIF)
