# Correction: Development and application of a *Bacillus anthracis* protective antigen domain-1 in-house ELISA for the detection of anti-protective antigen antibodies in cattle in Zambia

**DOI:** 10.1371/journal.pone.0211592

**Published:** 2019-01-25

**Authors:** Manyando Simbotwe, Daisuke Fujikura, Miyuki Ohnuma, Ryosuke Omori, Yoshikazu Furuta, Geoffrey Munkombwe Muuka, Bernard Mudenda Hang’ombe, Hideaki Higashi

The author contributions for Manyando Simbotwe are incomplete. The correct, complete contributions for Manayando Simbotwe are: Conceptualization, Data curation, Formal analysis, Investigation, Methodology, Writing: original draft, and Writing: review & editing.
