# Supplementary figures and images for: Development and application of a Bacillus anthracis protective antigen domain-1 in-house ELISA for the detection of anti-protective antigen antibodies in cattle in Zambia
Source: PLoS One. 2018 Oct 18;13(10):e0205986. doi: 10.1371/journal.pone.0205986 (PMC6193699; doi:10.1371/journal.pone.0205986)

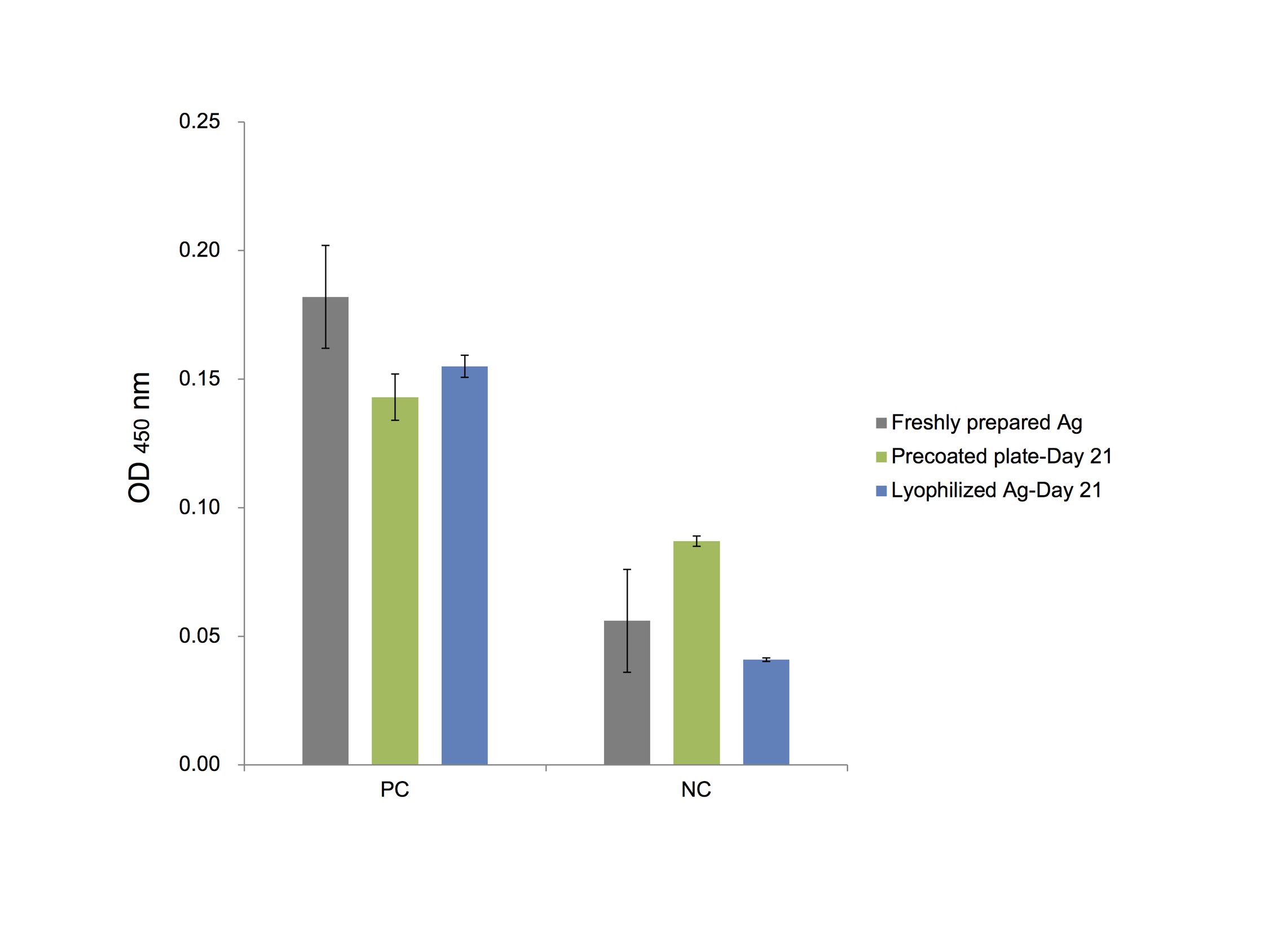

Supplement: S1 Fig — Comparison of the use of a freshly prepared ELISA plate using rPA-D1 stored at −80°C to that of a rPA-D1 precoated ELISA plate stored at RT and freshly prepared ELISA plate using lyophilized rPA-D1 stored at RT. Each PC and NC sample was measured in triplicate. The error bars represent the standard deviation. RT: Room temperature, PC: Positive control, NC: Negative control bovine serum. (TIF) [file pone.0205986.s001.tif]
